# Ganglion Cyst of the Peroneus Longus

**Published:** 2015-04-08

**Authors:** Andrew A. Marano, Paul J. Therattil, Dare V. Ajibade, Ramazi O. Datiashvili

**Affiliations:** Division of Plastic and Reconstructive Surgery, Department of Surgery, Rutgers New Jersey Medical School, Newark, New Jersey

**Keywords:** leg mass, ganglion cyst, peroneus longus, foot drop, peroneal nerve compression

**Figure:**
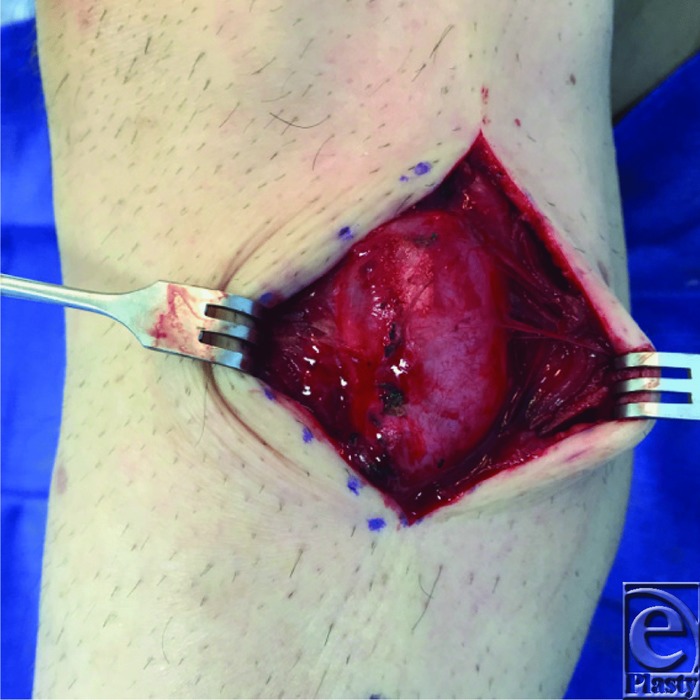


## DESCRIPTION

A 55-year-old man presented to the plastic surgery clinic with an enlarging mass of the left lateral leg over the head of the fibula. The patient reported paresthesia at the dorsum of the foot and had weakened dorsiflexion of the foot compared with the contralateral side.

## QUESTIONS

1. **What are ganglion cysts and where do they typically arise?**
2. **How does a ganglion cyst of the peroneus longus present?**
3. **What is the differential diagnosis for a cystic mass near the proximal tibiofibular joint?**
4. **What are the management options for this patient?**

## DISCUSSION

Ganglion cysts are benign myxoid cystic lesions that typically occur in the upper and lower extremities. Mucoid cystic degeneration occurs, likely secondary to trauma, as ganglia tend to arise in areas under constant mechanical stress.^1^ The periarticular soft tissues are most commonly involved, but ganglia can also occur in the tendon sheath, muscle, nerve, or periosteum. The most common locations for ganglion cysts are the hand and the wrist, followed by the ankle and the foot.^2^ The presentation of our patient is uncommon considering the location as well as the symptomatology demonstrated by the mass.

When arising from the peroneus longus muscle or tendon, ganglia often present with only swelling over the lateral aspect of the leg. Less commonly, peroneal nerve compression causes burning pain, numbness, or paresthesia of the lateral leg as well as weakness in foot dorsiflexion.^3^ When peroneal nerve compression is severe, patients may develop loss of sensation at the first dorsal webspace as well as foot drop.^4^ The typical course for a ganglion is cyclic and triphasic, with growth, plateau, and diminution stages. As the lesion grows, patients often feel discomfort or pain. These symptoms then resolve as the size of the lesion plateaus. Eventually the cyst diminishes in size, but refilling may occur.^4^

There is a broad differential for cystic lesions occurring near the proximal tibiofibular joint. The most common differential diagnosis is a synovial cyst, which is caused by an increase in intra-articular pressure secondary to synovitis or joint injury. This increase in pressure leads to outpouching and herniation of the joint capsule and formation of a cystic structure at the tibiofibular joint. Other possible causes include periarticular or intramuscular myxoma, cystic degeneration of a schwannoma or neurofibroma, or synovial sarcoma.^1^ Because these lesions share similar clinical presentations, imaging is a helpful adjunct. On magnetic resonance imaging, ganglia are delineated, round, lobulated fluid collections. They typically have low intensity on T1-weighted sequences and high intensity on T2-weighted sequences, with wall enhancement with intravenous contrast.^2^ Bony erosion may be apparent if the cyst is large enough, producing a false resemblance to a malignant lesion. If peroneal nerve entrapment occurs, denervated muscle may manifest as increased muscle signal.^1^ Histological analysis demonstrates a translucent mass with a dense fibrous connective tissue wall and focal areas of chronic inflammation.^5^

Ganglion cysts are typically treated with surgical excision. Surgical management of ganglia arising from the peroneus longus involves complete excision with careful preservation of the common peroneal nerve. If it is not possible to preserve the nerve and perform total excision, partial excision or marsupialization may be considered. It is important to identify any communication that may exist between the cyst and the tibiofibular or patellar joints, as failure to excise the ganglion stalk is a common cause of recurrence.^3^^,^^6^ Overall, ganglion cysts of the lower extremity have been reported to recur in approximately 10% of cases.^7^ Alternatives to surgery include injection of sclerosing agents following aspiration of the gelatinous contents of the ganglion, as well as radiotherapy.^8^

While ganglion cysts of the peroneus longus tendon are managed in a similar manner to other ganglion cysts, they may present with the added complication of peroneal nerve compression. For this reason, postoperative monitoring of strength and sensation is essential in patients whose initial presentation involved neurological deficits. In such cases, electromyography may be a helpful tool for assessing reinnervation.^8^

**Figure 1:**
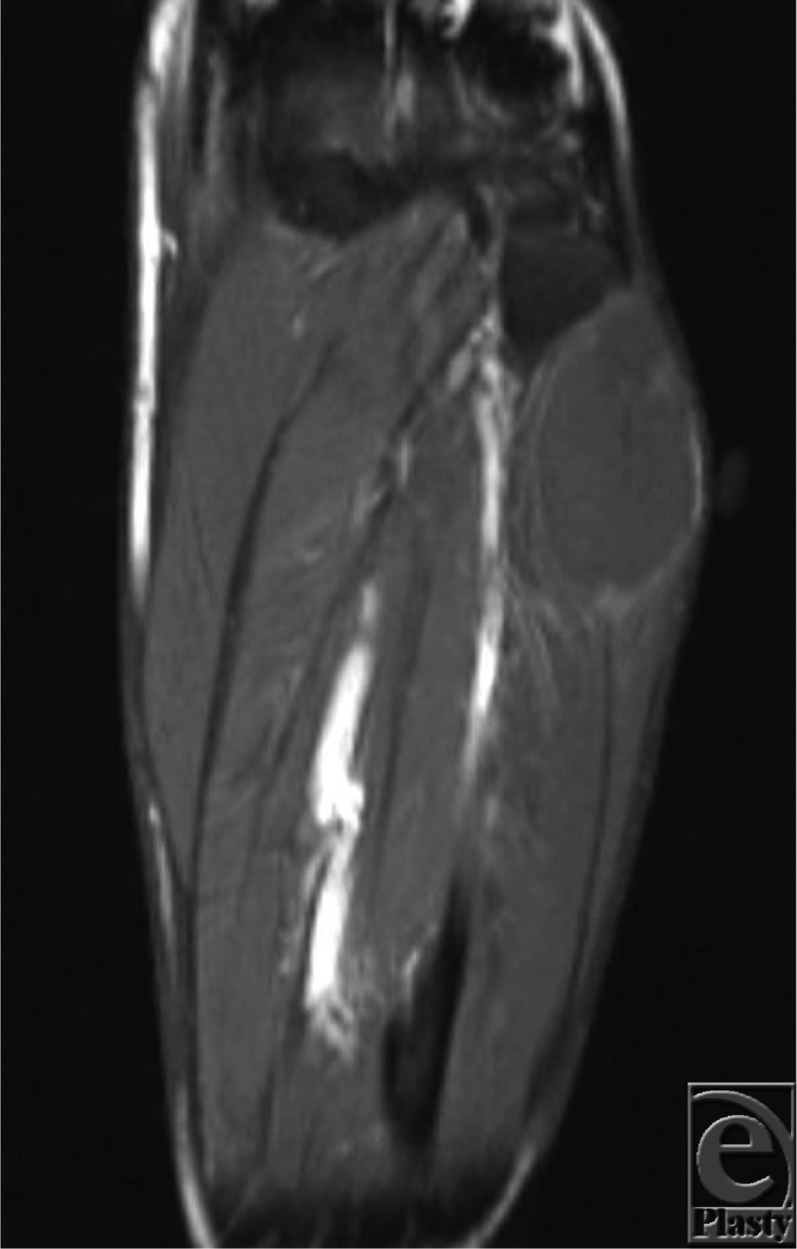
Coronal contrast-enhanced, T1-weighted magnetic resonance image of the peroneus longus ganglion cyst.

**Figure 2:**
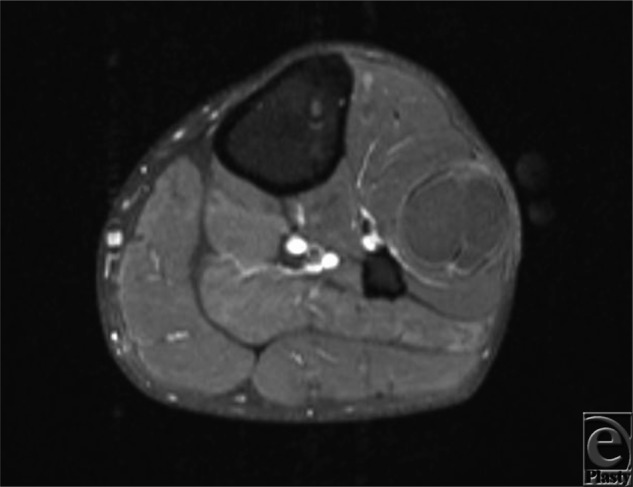
Axial contrast-enhanced, T1-weighted magnetic resonance image of the peroneus longus ganglion cyst, well-circumscribed.

**Figure 3:**
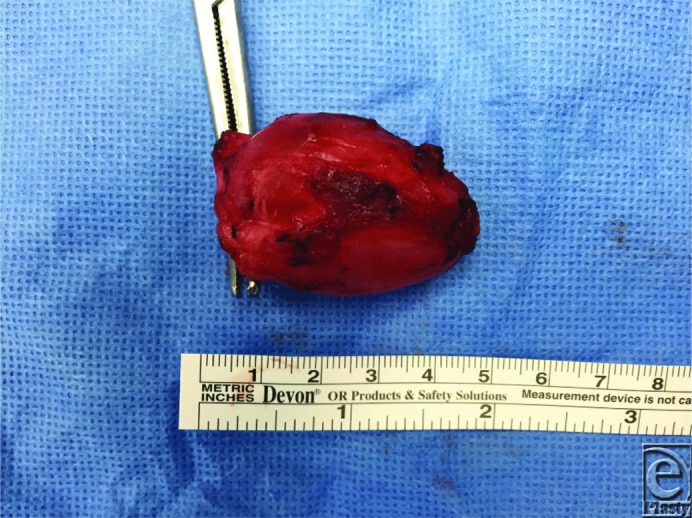
Intraoperative view of ganglion cyst of the peroneus longus.

After magnetic resonance imaging demonstrating a likely ganglion cyst (see Figs 1 and 2), our patient underwent excision of the mass under general anesthesia. A longitudinal incision was carried out over the mass and blunt dissection was used to isolate the ganglion. The mass was intimately involved with the peroneus longus muscle belly and had multiple lobular extensions. The stalks of the ganglion were dissected down to the tibiofibular joint and resected at this level to minimize risk of recurrence. The specimen was removed (see Fig 3) and the incision closed. Postoperatively, the patient reported resolution of his paresthesia and significant return in strength of foot dorsiflexion.
